# Lightweight neural network for smart diagnosis of cholangiocarcinoma using histopathological images

**DOI:** 10.1038/s41598-023-46152-6

**Published:** 2023-11-01

**Authors:** Shubhadip Chakrabarti, Ummity Srinivasa Rao

**Affiliations:** School of Computer Science and Engineering, Vellore Institute of Technology, Chennai Campus, Chennai, Tamil Nadu 600127 India

**Keywords:** Classification and taxonomy, Computational models, Data acquisition, Image processing, Machine learning

## Abstract

Traditional Cholangiocarcinoma detection methodology, which involves manual interpretation of histopathological images obtained after biopsy, necessitates extraordinary domain expertise and a significant level of subjectivity, resulting in several deaths due to improper or delayed detection of this cancer that develops in the bile duct lining. Automation in the diagnosis of this dreadful disease is desperately needed to allow for more effective and faster identification of the disease with a better degree of accuracy and reliability, ultimately saving countless human lives. The primary goal of this study is to develop a machine-assisted method of automation for the accurate and rapid identification of Cholangiocarcinoma utilizing histopathology images with little preprocessing. This work proposes *CholangioNet*, a novel lightweight neural network for detecting Cholangiocarcinoma utilizing histological RGB images. The histological RGB image dataset considered in this research work was found to have limited number of images, hence data augmentation was performed to increase the number of images. The finally obtained dataset was then subjected to minimal preprocessing procedures. These preprocessed images were then fed into the proposed lightweight *CholangioNet*. The performance of this proposed architecture is then compared with the performance of some of the prominent existing architectures like, VGG16, VGG19, ResNet50 and ResNet101. The Accuracy, Loss, Precision, and Sensitivity metrics are used to assess the efficiency of the proposed system. At 200 epochs, the proposed architecture achieves maximum training accuracy, precision, and recall of 99.90%, 100%, and 100%, respectively. The suggested architecture's validation accuracy, precision, and recall are 98.40%, 100%, and 100%, respectively. When compared to the performance of other AI-based models, the proposed system produced better results making it a potential AI tool for real world application.

## Introduction

Cancer, one of the deadliest ailments, constitutes multi-dimensional malignancies in different parts of human body and eventually leads to death, especially if it is not treated promptly at its early stage. Medical treatment involves careful detection and diagnosis. Cholangiocarcinoma, a type of cancer that develops in the cells of the bile duct, which plays an important function in the human digestive system. This malignancy is often caused by alterations in the DNA of bile duct cells.

An anatomical perspective^1^ of cholangiocarcinoma, which happens to be the second most common primary hepatic malignancy, reveals that it may be either intrahepatic cholangiocarcinoma (iCCA) or extrahepatic cholangiocarcinoma (eCCA), depending on the location of the affected area in the biliary tree. Bile, a vital digestive juice, is produced in the liver and delivered to the small intestine via the bile duct. Intrahepatic bile ducts are made up of ductiles inside the liver and consist of the left and right hepatic ducts. These two primary ducts exit from the liver and connect to the common hepatic duct in extrahepatic region known as the hilum. The common hepatic duct connects with the cystic duct, which emerges from the gall bladder, to produce the common bile duct, which flows to the duodenum after passing through the pancreas and connecting with the pancreatic duct at the ampulla of vater. Thus, extrahepatic cholangiocarcinoma (eCCA) involves perihilar bile duct or distal bile duct.

Cholangiocarcinoma appears to be typically incurable and fatal. It is responsible for nearly 15–20 percent of primary hepatobiliary carcinoma and has been shown an upward trend all over the world. It is significant that Southeast Asia has become the region that has been hit the most, with an annual incidence that may reach as high as 71.3 cases per million inhabitants^2^. In wealthy nations, the mortality rate owing to Cholangiocarcinoma may be less than 2, while in developing nations, such as Thailand, South Korea, and China, the rate of mortality due to Cholangiocarcinoma has been documented as being more than 6 per millions of people. Even after five years, the usual survival rate is lower than fifty percent. The rate of their incident is increasing It is therefore imperative that early detection of Cholangiocarcinoma is of paramount importance to save precious human lives.

Conventional methods^3,4^ for diagnosing the illness include procedures such as ultrasound, computed tomography, magnetic resonance imaging, brush cytology, and forceps biopsy. However, the identification of cholangiocarcinoma has become extremely difficult due to the presence of multiple factors. For instance, the reduced detection sensitivity of CT, MRI, and ultrasound imaging might be associated to the progression of Cholangiocarcinoma in a longitudinal direction down the bile duct rather than in a radial direction. In addition, these methods are not appropriate for detecting Cholangiocarcinoma and its extent because of the interference caused by a large number of other variables related to physical illness. The brush cytology technique has become an increasingly prevalent tissue sample method for the diagnosis of cancer in the bile ducts. However, the system's sensitivity can only be adjusted within a range of 30–60% at the most. However, by utilising a different method known as the forceps biopsy, it may be improved to anywhere between 43% and 81%^5^. In point of fact, the many traditional medical diagnosis tools that have been developed over time have substantial limitations in terms of both their sensitivity and their accuracy in the identification of Cholangiocarcinoma. This necessitates automation intervened enabling modality for precise detection of bile duct malignancy at an early stage to ensure saving of numerous humans’ lives.

The major goal is to create a reliable and efficient AI-based diagnostic tool for Cholangiocarcinoma. The following are the significant contributions of this paper in accomplishing the primary goal of building the most efficient and highly reliable AI-based system for the detection of Cholangiocarcinoma:

- Using RGB images from a histopathology image dataset that was procured from Kaggle, the current effort intends to construct an AI-based system for the detection of cholangiocarcinoma.
- The authors^6^ of this dataset of images based on Cholangiocarcinoma demonstrated how HSI data may be used to create more accurate AI models for disease diagnosis. It is crucial to remember that the procurement and development of such HSI image data are costly, difficult, and time-consuming. As a result, producing HSI data for diagnostic purposes might not always be possible. Therefore, it becomes urgently necessary to develop an accurate AI-based system that uses RGB images for the diagnosis of Cholangiocarcinoma.
- The current study entails data augmentation of the available RGB-based Histopathological images in order to support the extension of image data instances for the creation of a more all-encompassing, effective, and dependable AI-based system.
- For the purpose of diagnosing Cholangiocarcinoma, we created the neural network *CholangioNet*. The creation of an effective and more trustworthy AI-based system for the diagnosis of cholangiocarcinoma would be made possible by this suggested neural network. With maximum training accuracy, precision, and recall recording of 99.90%, 100%, and 100% respectively, and maximum testing accuracy, precision, and recall recording of 98.40%, 100%, and 100% respectively, this architecture has been proven to produce excellent results.
- We compare the performance of the proposed neural network architecture with that of well-known existing architectures like VGG16, VGG19, ResNet50, and ResNet101 as well as with the current state of the art to acquire a deeper understanding of the accomplishments of the suggested *CholangioNet*. Following analysis, it was discovered that the proposed network outperformed all current architectures and state-of-the-art methods, demonstrating it better capability in producing more accurate results while diagnosing Cholangiocarcinoma. As a result, it has been confirmed that the suggested Cholangiocarcinoma is a highly effective and trustworthy AI-based model for the diagnosis of Cholangiocarcinoma.

The remainders of the paper are as follows: The second section is a comprehensive literature review that focuses on contemporary Artificial Intelligence-based techniques for Cholangiocarcinoma diagnosis in particular. This section also describes the constraints of automated medical diagnostic procedures currently in use. This section concludes with a summary of the literature review conducted for this study. In the third section, an analysis on the dataset utilized for this investigation is performed. It also clarifies why the research and development of AI-based models that use RGB images for diagnosis rather than Hyperspectral images is of the uttermost importance. This section also explains the various image preprocessing techniques utilized in this investigation. In addition, we also describe the architecture of the proposed *CholangioNet*. Using transfer learning, the performance of the proposed architecture was compared to the performance of several prominent existing architectures. In Sect. "Results and discussion", we began with discussing the environment in which our models were formulated. The metrics used to evaluate the efficacy of AI-based models in this study are also noted. In conclusion, we analyze the usefulness of each model studied throughout this investigation. In addition, relevant diagrams and tables are attached as exhibits. Finally, the performance of the proposed model was compared to that of existing state-of-the-art models and analyzed. Lastly, Section "Conclusion and future work" gives the conclusion and thoughts for the future scope of this research work.

## Literature survey

Various Artificial Intelligence based Machine Learning (ML) and Deep Learning (DL) techniques have been introduced and used in the diagnosis of Cancer, specifically Cholangiocarcinoma or Bile Duct Cancer, to successfully analyse tumorous and non-tumorous histological images. The following are some of the computer vision approaches utilised for histopathology image analysis.

Li Sun^6^ et al., proposed a methodology to diagnose Cholangiocarcinoma using Microscopic Hyperspectral Pathological dataset. To begin with, they developed an image dataset from 174 clinically microscopic slides of bile duct tissue. They could effectively generate 880 scenes. They essentially captured the images in two formats namely, in 3-channel format (RGB image) and in 30-channel format (Hyperspectral image). They essentially aimed at exploiting the power of HSI data for automating the diagnosis of cholangiocarcinoma using Histopathological images. They showed that using HSI data could result in slightly better performance of the model in comparison with RGB image. The models ResNet and InceptionNet was found to record a training accuracy of 85% and 85.3% respectively. To further enhance the performance of the model, data mining from the HSI using spectral interval convolution and normalization schemes was also done. These enhancements resulted in 88.2% and 87.9% accuracy from ResNet50 and InceptionV3 respectively. Though Hyperspectral image data can generate better diagnostic results, however procuring Hyperspectral image data for diagnosis may not always be feasible. Hence, models with better diagnostic results using RGB image also needs to be constructed.

Uyumazturk, B et al.^7^, developed a web-based AI assistant and demonstrated how the AI-based diagnostic system could strongly bias the performance of expert’s diagnosis while diagnosing HCC and Cholangiocarcinoma. The authors used 70 WSI diagnostic slides for the development of the deep learning-based AI model. They specifically used DenseNet121 for the development of the AI Model. The deep learning model used in this study achieved an internal validation accuracy of 88.5%. It also recorded 84.2% accuracy on an independent test set. When the model was deployed as an AI assistant on a web-based interface, the pathologists were found to strongly get biased by the results generated by the AI model. It was found that when the AI model gave correct results, it significantly improved the performance of the pathologist. However, when the model was found to be generate incorrect results, it resulted in reduced performance of the pathologists. This indicated that though it was important to develop AI-based models for significant improvement in the accuracy of the diagnostic results, AI model was also capable of introducing strong negative biases during diagnosis. This highlights the importance of designing an AI model with the highest performance to make the AI -based system more reliable.

Chunmei Yang et al.^8^, developed two models using Random Forest Algorithm for the detection of Lymph Node Metastasis (LNM) and Differentiation Degree (DD) of Extrahepatic Cholangiocarcinoma using Magnetic Resonance Imaging (MRI) Images. The authors used MaZda software to first extract 300 features from each ROI. By utilising the ADC values of tumors and the radiomics signatures, a radiomics model was constructed utilising the Random Forest method to predict DD (model A) and LNM (model B). It was found that model corresponding to the detection of Differentiation Degree recorded an average accuracy of 69.9% and 71% on training and testing sets respectively. The average sensitivity and specificity were found to be 70.7% and 70.6% on the training set, while it was found to be 73.2% and 68.8% on testing set respectively. On the other hand, the model corresponding to the detection of Lymph Node Metastasis was found to record an average accuracy of 81.4% on the training set and 83.3% on the testing set. The average sensitivity and specificity were found to be 80.1% and 82.6% on the training set, while it was found to be 85.4% and 81.8% on testing set respectively. As understood from^7^, though the performance achieved is good, however, additional performance improvements are required to make the AI-based system more reliable for real-world applications.

Jiarong Zhou et al.^9^, used a total of 616 nodules for detection and classification of focal liver lesions. These nodules were predominantly composed of malignant lesions and benign lesions. Intrahepatic Cholangiocarcinoma, Hepatocellular Carcinoma and Metastasis were the three malignant lesions whereas the cyst, focal nodular hyperplasia and hemangioma were the three benign lesions included in the study. Three different 2.5D detection networks and one 3D classification network were used to develop this AI based system. Average precision, recall, and F1 score for detecting focal liver lesions using the proposed model were 82.8%, 93.4%, and 87.8%, respectively. The proposed model for binary classification achieved an average accuracy of 82.5% while for multiclass classification it achieved an average accuracy of 73.4%. In spite of the fact that the suggested architecture has the potential to produce encouraging results, there is still room for improvement in terms of the diagnostic accuracy; this might lead to the creation of an AI-based diagnostic system that is more trustworthy. D. Selvathi et al.^10^, used CT images to automate the classification and segmentation of liver tumor. They initially processed the images by segmenting them using adaptive thresholding and morphological operations, followed by extraction of the tumor region using Fuzzy C Means. They then applied Contourlet transform to obtain the statistical information from the images. The finally processed images were then fed into the Extreme Learning Machine (ELM) Classifier to enable classification. Using Adaptive Thresholding and Fuzzy C Means, they achieved liver tumour segmentation accuracy of 97.72% and 98.7%, respectively. They also noted that the proposed ELM method recorded an accuracy of 96.56%, and sensitivity, and specificity of 94.33% and 97.78% respectively, which was found to be higher than the conventional methods. Though the results obtained are good, however, a further improvement in the performance of the model will make it more reliable. As understood from^7^, though the performance achieved is good, however, additional performance improvements are required to make the AI-based system more reliable for real-world applications.

Abhishek Midya et al.^11^, used the data procured from 223 patients ailing with Hepatocellular Carcinoma (HCC) and Intrahepatic Cholangiocarcinoma (ICC) to classify images into its respective categories. A deep learning model by modifying the pre-trained Inception Network was proposed in this study. The proposed model recorded an AUC score and accuracy of 0.72, 69.70%. The specificity and sensitivity, recorded 44% and 100% respectively. Though the proposed architecture is capable of generating good results, however a further improvement in the diagnostic accuracy will result in the development of a more reliable AI based diagnostic system. Donlapark Ponnoprat et al.^12^, used two image datasets to automate the classification of CT images into HCC and ICC. During the first phase of the study, the images were segmented using modified U-Net and CRF post processing. Later, the features from the discriminative regions are extracted by analyzing the histograms of the segmented images. These features were given as an input to the Support Vector Machine (SVM) Model for classification. The proposed algorithm could record a recognition accuracy of 88% on the private dataset used in this study. Although the suggested architecture has the potential to yield great results, a more trustworthy AI-based diagnostic system may be developed by increasing diagnostic precision even more. Hirotsugu Nakai et al.^13^, presented a study to classify liver cancer into poorly differentiated HCC (pHCC), ICC and moderately differentiated HCC (mHCC). Initially, the tumor images were manually cropped out using RectLabel software. Cropping resulted in 9 images from each case. These images were then scaled down to (70,70). Finally, the pixel values of the image were transformed so that the mean remained zero and the standard deviation stayed one. Later, one input CNN model (with no tumor marker information) and 2-input model (with tumor marker information) was developed. The accuracy rates of the one input and two input models were 60% and 61%, respectively, which was greater than the radiologists accuracy rates. It was also observed that the two-input model displayed higher specificity than radiologists for classifying HCC into mHCC and pHCC. It has been shown that the suggested design may generate passable results, but additional advancements in diagnostic performance can enable the generation of more precise and rapid diagnostic findings.

Charlie A. Hamm et al.^14^, proposed a deep learning architecture for the diagnosis of Liver Tumor. The system utilized 494 lesions on Multiphase MRI from 6 different hepatic tumor categories for this task. They divided the dataset into training and testing sets and then performed data augmentation to increase the number of training instances. Monte Carlo Cross Validation technique was also performed. The proposed model recorded an accuracy of 92%. It also recorded a sensitivity of 92% and specificity 98%. When the model was tested on random unseen instances, it recorded a sensitivity and specificity of 90% and 98% respectively. The performance of the model was finally compared with the diagnostic accuracy achieved by the radiologists. The radiologist’s displayed an average sensitivity and specificity of 82.5% and 96.5% respectively on the same instances. They also noted a 90% sensitivity, 93.5% true positive rates and 1.6% false positive rates for classifying HCC. The results showed that the model did a better job of interpreting the MRI scans than people did, it is possible that the efficiency of the AI system might be improved by enhancing the model's performance. This appears to be the case despite the fact that the model outperformed humans. Clinton J. Wang et al.^15^, proposed an interpretable deep learning system. This study is an extension of the work done in^14^. This system labelled a subset of each image class. The presence of these features was inferred by an algorithm by analyzing the activation patterns of the pre-trained models. Feature maps for these images were generated. Further, they also added the relevance score to each of the feature identified in the image. This system recorded an 82.9% sensitivity. The results show that the proposed design may produce good results, while diagnostic performance should be enhanced to make diagnoses faster and more accurate. Bulat Ibragimov et al.^16^, proposed a universal system that could identify critical radiosensitive region that are important to be left during a liver stereotactic body radiation therapy (SBRT). They investigated if a CNN model could associate CT images and Radiation Therapy (RT) dose plans with post RT toxicities without the use of segmentation masks. The CNN was also made to understand the RT plans. This resulted in the generation of risk maps that were taken up to compare with the existing clinical studies. It was observed that though the prediction accuracy was found to be moderate, the model could recognize the radiosensitive regions quite accurately. Looking at the results obtained, they also noted that the model was able to discover the critical dose patterns quite well. Although the evidence suggests the proposed architecture has the potential to yield serviceable outcomes, further improvements in diagnostic performance hold the promise of enabling the production of speedier diagnostic conclusions.

Hongpeng Chu et al.^17^, presented a study on radiomics for the prediction of futile resection in ICC. They used the data collected from 203 ICC patients. The clinical information was extracted using Random Forest to develop a clinical model. Similarly, logistic models were used to select the radiomics features for the development of the radiomics model. The developed radiomics signature and clinical risks were used to develop a combined model. The Radiomics model was found to record AUC comparable as that of the radiomics model. However, it recorded a higher AUC in comparison with the clinical model. The Radiomics model achieved 78.7% accuracy, 84.6% sensitivity, and 77.1% specificity, respectively. The Logistics Model, on the other hand, achieved 59% accuracy, 69.2% sensitivity, and 56.2% specificity. The accuracy, sensitivity, and specificity of the Combined Model were 75.4%, 92.3%, and 70.8%, respectively. Though the results seem promising, a further enhancement in the performance of the AI system will make the system more reliable. Qiyuan Wang et al.^18^, used CT images procured from 234 individuals to devise an approach to detect and classify ICC and HCC. The procured images were categorized into three classes, namely HCC, ICC and Normal. They proposed a novel Siamese Cross Contrast Neural Network (SCCNN) in this study. They designed SCCNN extends the concepts of CCNN with Siamese technique and gradient-stop MLP classifiers. This enabled the model to behave like a normal CNN. Discriminative Information Based Similarity (DisIBS), a new IBS based measurement is also proposed in this study. The highest accuracy recorded for three class classification corresponds to 90.22% for slice and 94.92% for patient level. While the highest accuracy recorded for two class classification corresponds to 94.17% and 97.44% for slice and patient level respectively. Even though the proposed architecture is capable of producing promising results, a further improvement in diagnostic performance is absolutely necessary to enable the generation of more reliable and faster diagostic results. Zhang Jun et al.^19^, proposed an algorithm to differentiate Combined Hepatocellular and Cholangiocarcinoma (CHC) and ICC using radiomics. The proposed AI model was developed on 86 patients. The radiomics features of enhanced CT from 12 different ROIs were used to build the radiomics score. Though the model was found to display better performance than human interpretation of the MRI images, however, a further enhancement in the performance of the model could make the AI system more reliable.

Rajasvaran Logeswaran^20^, used Magnetic Resonance Cholangiopancreatography (MRCP) images to automate the detection of Cholangiocarcinoma. The authors developed an Artificial Neural Network (ANN), namely the Multilayer Perceptron (MLP) to differentiate the images into their respective classes. The developed model could classify the normal and tumor images with 94% accuracy on the test set of the image dataset. Upon further analysis they found that they were also able to detect the tumor images from a large pool of images containing common biliary disease with 88% accuracy. The findings reveal that the recommended design may generate satisfactory results, although diagnostic performance may be improved to make diagnoses faster and more accurate. Hanyue Xu et al.^21^, proposed an algorithm to differentiate ICC and Hepatic Lympoma (HL) using textural parameters. They explored various combinations of feature selection models and classification models to determine the most optimal AI model in distinguishing ICC and HL in CT images. They used 10-fold cross validation during the development of this AI system. The combination of Random Forest and Linear Discriminant Analysis (LDA) produced the best results recording a 96.9% accuracy and an AUC score of 0.997. The results show that the design that was suggested may produce acceptable results, but diagnostic performance could be improved to make evaluations faster and more accurate.

Koichiro Yasaka et al.^22^, proposed a CNN model for classifying the liver masses into five different categories in CT images. The JPEG images obtained were cropped and resized before finally feeding them into the proposed model for supervised training. The median accuracy on the testing set is determined to be 84% The median AUC score to distinguish between A–B and C–E is found to be 0.92. Preliminary results show promise for the suggested design to yield adequate outcomes, and improvements to diagnostic performance in the future hold the possibility of enabling the creation of more accurate and faster diagnostic conclusions. Liu X et al.^23^, designed a system to automate the detection and classification of Cholangiocarcinoma (cHCC-CC), Cholangiocarcinoma and Hepatocellular Carcinoma (HCC) in MRI and CT images. Tumor Segmentation was performed to extract 1419 features. PCA was also done to choose the 20 most important features. They finally used SVM as the classifier. MRI radiomics features was found to display better AUC score (highest AUC score: 0.77) in comparison with the CT radiomics features. While there is some preliminary evidence that the proposed design may produce results that are satisfactory, there is still room for future improvements to diagnostic performance, which have the potential to make it possible to generate diagnostic conclusions that are both more accurate and quicker. Lei Xu et al.^24^, proposed an algorithm to predict the Lymph Node (LN) status in ICC patients. The features best used to detect LN status were selected using maximum Relevance Minimum Redundancy (mRMR) algorithm. These features were used to develop a SVM model. SVM score was calculated for each image being taken into consideration. A combination nomogram using the clinical features and the SVM score was developed. The combination nomogram was found to generate better prediction results than SVM alone. However, additional advancements in diagnostic performance hold the possibility of enabling the generation of both more accurate and quicker diagnostic findings, even though preliminary data shows the suggested architecture has the ability to produce serviceable results.

B. Ashreetha et al.^25^, proposed a system to segment liver and tumor regions from an abdominal image. They initially extracted the pixel level features from the abdominal CT images using Gabor filters. These filtered images are then trained on Random Forest**,** Support Vector Machine and Deep Neural Network to enable the segmentation of liver from an abdominal CT image. The pixel level features are again extracted from these segmented CT images using Gabor filters. Finally, they trained these images on Random Forest**,** Support Vector Machine and Deep Neural Network to enable the segmentation of tumor from an liver image. They finally determined that the Random Forest was found to perform better than the other classifiers taken into consideration in their study. The findings indicate that the proposed design may yield satisfactory outcomes, however diagnostic performance should be enhanced to speed up and better characterise assessments. Xiaoliang Xu et al.^26^, proposed a system for classification of HCC and ICCA on CT scan images. The radiomic features were extracted from the CT scans. The features selected were then carried out using LASSO logistic method. On the training set, the SVM model was found to record a 0.855 AUC score. While on the testing set, the model was found to record 0.847 AUC score. Though the performance of the proposed system is good, however, a further enhancement in the performance of the model is necessary to make the AI based diagnostic system more reliable. Srilatha Tokala et al.^27^, proposed a system for liver disease prediction and classification using ML techniques. The dataset used in their study is first preprocessed. The preprocessed dataset is then subjected to feature selection techniques to obtain features important for this prediction. This enabled enhancing the prediction capability of liver disease. The features were then fed into Logistic Regression, Support Vector Machine, K Nearest Neighbors and Random Forest classifiers. Random Forest was found to record the highest accuracy as 88%. Though the performance of the ML classifiers were found to be satisfactory, the use of more reliable models for disease diagnosis becomes important for better impact in this sector. Meiling Wang et al.^28^, proposed a novel Deep Margin Cosine Autoencoder (DMCA) model for Medical based Hyperspectral images (MedHSI). They have initially used a deep autoencoder to extract the features from the MedHSI. The features are then fed into the softmax classifier to predict the results. Further, the concept cosine margin is also introduced in this study. A two stage training strategy is proposed to train the developed DMCA model. Though the performance of the proposed system is quite satisfactory, a further improvement will make this AI based system more dependable and thus more popular in this field of medical industry.

S. Mahmoudi et al.^29^, proposed a system to differentiate between iCCA and HCC using CT images. They used the combination of radiomics features and clinical features to classify the patients into their respective categories. They used logistic regression classifier to achieve this purpose. They could attain the maximum ROC of 0.82 using this classifier. Though the performance of this proposed system seems promising, however, a further improvement in the diagnostic performance is desirable as this would increase the reliability of this AI model, making it an AI based system potential enough for real world deployment in the medical industry. Jiong Li et al.^30^, proposed a system for the prediction of angiogenesis in eCCA in MRI images. They initially extracted the features from T1 weighted, T2 weighted and diffusion weighted images using MaZda software. After the determination of the important features, these features were used to predict VEGF expression using a classification model and predict MVD using a regression model. Among all the combination of classifiers and proprocessing techniques that were taken into consideration during their study, they discovered the combination of Z score standarization and Logistic regression model to display excellent results recording an average AUC score of 0.912 and 0.884 on training and testing cohorts respectively. On the other hand, the Z score standarization in combination with Stochastic gradient descent based linear regression was seen to perform better, recording an average ajusted R^2^ of 0.975 on the training cohort. Though the machine learning models were displaying good results, however a further improvement in the performance of the AI based diagnostic model can lead to the development of a more dependable AI model for practical application. Taiichi Wakiya et al.^40^, proposed a deep learning based system that could predict post operative occurance of Intrahepatic Cholangiocarcinoma using CT images. The authors collected images from 41 patients. The accuracy, specificity and sensitivity on the validation set of the dataset recorded 96.5%, 94% and 97.8% respectively. Further, the ROC was also determined to be 0.994. The findings indicate that the proposed design has the potential to yield satisfactory outcomes. However, there remains an opportunity for further enhancement in diagnostic performance, potentially enabling faster and more accurate diagnoses in the future.

Table 1 summarizes the algorithms/models used in the existing state of art as discussed above. The limitations of each state of the art is also highlighted.

**Table 1:** Summary of the existing state of the art.

Research paper	Models/Algorithms used	Limitations
6	ResNet50 and InceptionNet	Though Hyperspectral image data can generate better diagnostic results, however procuring Hyperspectral image data for diagnosis may not always be feasible. Hence, models with better diagnostic results using RGB image also needs to be constructed
7	DenseNet121	The AI model achieved 88.5% accuracy on the internal validation sets and 84.2% on the independent test sets When the prediction was correct, the assistance improved the accuracy across all pathologist experience levels, however, when the model was found to be incorrect, the assistance significantly decreased accuracy across all pathologist experience levels for all case difficulty levels This highlights the importance of designing an AI model with the highest performance to make the system more reliable
8	The ADC values of tumours and the radiomics signatures were used to create a radiomics model using the Random Forest method to predict DD (model A) and LNM (model B)	As understood from^7^, though the performance achieved is good, however, additional performance improvements are required to make the AI-based system more reliable for real-world applications
9	Three 2.5D Faster RCNN w/ FPN detection networks One 3D ResNet18 classification network	In spite of the fact that the suggested architecture has the potential to produce encouraging results, there is still room for improvement in terms of the diagnostic accuracy; this might lead to the creation of an AI-based diagnostic system that is more trustworthy
10	Extreme Learning Machine (ELM) classifier	According to^7^, even though the obtained performance is excellent, additional performance enhancements are necessary to make the AI-based system more reliable for real-world applications
11	Modified InceptionNet	Even though the proposed architecture is capable of producing promising results, a further improvement in diagnostic performance is absolutely necessary to enable the generation of more reliable and faster diagostic results
12	U-Net, Support Vector Machine (SVM) and Local Binary Pattern (LBP)	Although the suggested architecture has the potential to yield great results, a more trustworthy AI-based diagnostic system may be developed by increasing diagnostic precision even more
13	One Input Model (without Tumor information) and Two input model (with tumor information)	It has been shown that the suggested design may generate passable results, but additional advancements in diagnostic performance can enable the generation of more precise and rapid diagnostic findings
14	Convolution Neural Network	The results showed that the model did a better job of interpreting the MRI scans than people did, it is possible that the efficiency of the AI system might be improved by enhancing the model's performance. This appears to be the case despite the fact that the model outperformed humans
15	Proposed an interpretable deep learning system	The results show that the proposed ML algorithms may produce good results, but the diagnostic performance should be enhanced to make diagnoses faster and more accurate
16	Convolution Neural Network	Although preliminary evidence suggests the proposed architecture has the potential to yield serviceable outcomes, further improvements in diagnostic performance hold the promise of enabling the production of both more accurate and speedier diagnostic conclusions
17	Random Forest Algorithm, Logistic Model and Combined Logistic Model	While preliminary evidence suggests that the proposed design has the potential to produce satisfactory results, future diagnostic performance enhancements hold the promise of enabling the generation of diagnostic conclusions that are both more accurate and faster
18	Novel Siamese Cross Contrast Neural Network (SCCNN)	There is still room for improvement in terms of the diagnostic accuracy; this might lead to the development of an AI-based diagnostic system that is more reliable. In spite of the fact that the recommended architecture has the ability to deliver positive results, there is still room for performance improvement in this area
19	An algorithm to differentiate Combined Hepatocellular and Cholangiocarcinoma (CHC) and ICC using radiomics	The results show the suggested design may produce adequate results, but there is still room for development in terms of diagnostic performance, which might lead to quicker and more precise diagnoses in the future
20	Multi-Layer Perceptron (MLP)	The findings reveal that the recommended design may generate satisfactory results, although diagnostic performance may be improved to make diagnoses faster and more accurate
21	Used 9 classification models and 5 feature selection models	The results show that the design that was suggested may produce acceptable results, but diagnostic performance could be improved to make evaluations faster and more accurate
22	Convolution Neural Network	Preliminary results show promise for the suggested design to yield adequate outcomes, and improvements to diagnostic performance in the future hold the possibility of enabling the creation of more accurate and faster diagnostic conclusions
23	SVM classifier	While there is some preliminary evidence that the proposed design may produce results that are satisfactory, there is still room for future improvements to diagnostic performance, which have the potential to make it possible to generate diagnostic conclusions that are both more accurate and quicker
24	A combination nomogram using the SVM score, and clinical features was developed	However, additional advancements in diagnostic performance hold the possibility of enabling the generation of both more accurate and quicker diagnostic findings, even though preliminary data shows the suggested architecture has the ability to produce serviceable results
25	Random Forest (RF), Support Vector Machine (SVM) and Deep Neural Network (DNN)	The findings indicate that the proposed design may yield satisfactory outcomes, however diagnostic performance should be enhanced to speed up and better characterise assessments
26	Support Vector Machine (SVM)	It has been shown that the suggested design may generate passable results, but additional advancements in diagnostic performance can enable the generation of more precise and rapid diagnostic findings
27	Logistic Regression, Support Vector Machine, K Nearest Neighbors and Random Forests	The results show that the proposed ML algorithms may produce good results, but the diagnostic performance should be enhanced to make diagnoses faster and more accurate
28	Deep Margin Cosine Autoencoder	The findings reveal that the recommended design may generate satisfactory results, although diagnostic performance may be improved to make diagnoses more accurate
29	Logistic Regression	Though the performance of this proposed system seems promising, however, a further improvement in the diagnostic performance is desirable as this would increase the reliability of this AI model, making it an AI based system potential enough for real world deployment in the medical industry
30	Logistic Regression for classification and Stochastic Gradient Descent based linear regression for regression	Though the machine learning models were displaying good results, however a further improvement in the performance of the AI based diagnostic model can lead to the development of a more dependable AI model for practical application
31	ResNet50	The findings indicate that the proposed design has the potential to yield satisfactory outcomes. However, there remains an opportunity for further enhancement in diagnostic performance, potentially enabling faster and more accurate diagnoses in the future

## Dataset description

### Dataset description

The study uses a cholangiocarcinoma based histopathological image dataset^6^ procured from Kaggle. The image dataset has 880 images procured from 174 individuals. The image dataset comprises of 3 classes. Of the 880 scenes, 49 scenes are samples with full of cancer areas, 689 scenes are samples with part of cancer areas and 142 scenes are samples without cancer areas. This image database has both microscopy hyperspectral images and RGB images of Cholangiocarcinoma. The authors of this dataset noted that most of the times, such types of databases either have gray scale images or RGB images. It was also noted that such image types had limited information which restricted the performance of most of the deep learning models. Hence, hyperspectral images were introduced in the database.

**Figure 1:**
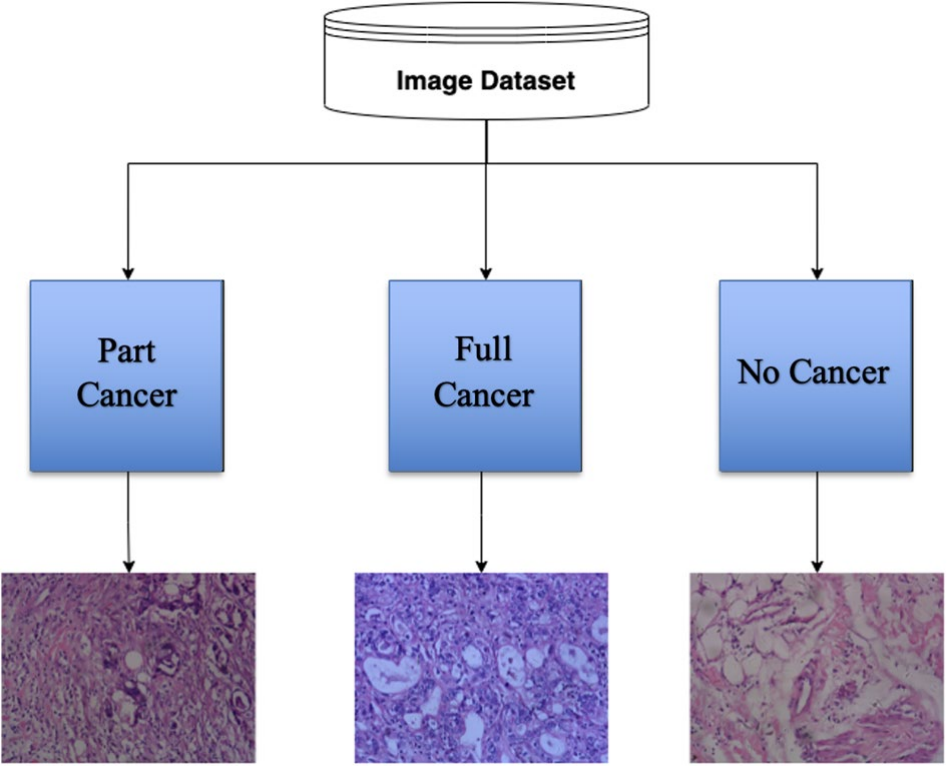
Image Dataset Structure. One image from each class present in the image dataset considered in this study is represented in this figure.

However, during the present work, it is noted that the generation of such hyperspectral images are costly, complex and time consuming. Fast Computers, Large Data Storage Capacities and Sensitive Detectors are needed for analyzing such hyperspectral data. Since, it is not always possible to have such huge computational resources, the development of efficient deep learning models for more accurate RGB image-based diagnosis would have impact on the greater part of the society in the current scenario. Hence, the present work essentially deals with development of a reliable AI based system for automating the diagnosis of Cholangiocarcinoma using RGB Histopathological images. Figure 1 shows one sample image from each class of the taken dataset.

### Preprocessing

Initial examination of the image dataset revealed that it contains 880 RGB photos from three distinct classes. The 880 scenarios are divided into 49 samples with entire cancer regions, 689 samples with partial cancer areas, and 142 samples without cancer spots. This suggested that the picture dataset has a class imbalance. Upon further examination, the images in this dataset was found to have a (1728, 304) size. We also discovered that the picture dataset has a restricted amount of images. As a result, offline data augmentation^38^ was conducted on the original RGB picture dataset to enhance the number of image samples from each class. It was also ensured that each class had a consistent quantity of picture samples. This would allow us to overcome the issue of class disparity.

The rotational range and shear range are set to 20 and 1.5, respectively, when doing offline data augmentation. The height and width shift ranges are set to 0.1 and 0.1, respectively. These variables might cause variation in the type of histopathological pictures produced. Horizontal and vertical flips were also permitted to increase the diversity of the pictures produced. Because RGB histopathological images are not always captured under the same lighting conditions, a brightness range of 0.5 to 1.5 is included. This procedure also allows for ZCA whitening. All of these features would allow for the construction of more realistic photos, allowing for the building of an AI model whose performance would be unaffected by photographs recorded under various situations. This would lead to the creation of a more general and dependable AI-based approach. Table 2 shows the photos that were created after each parameter was applied during data augmentation.

**Table 2:** Sample images generated after the application of each parameter during Data Augmentation.

Parameter	Sample image
Actual image	
Rotational range	
Shear range	
Width shift range	
Height shift range	
Horizontal flips	
Vertical flips	
Brightness range	

A total of 5000 photos from each class were produced in the final image dataset, which had a total of 15000 images. The current work then makes additional use of this enriched image dataset. Next, various image preprocessing techniques were applied to these images from the image dataset.

Image enhancing techniques are first applied to the photos from the final dataset. The photos are initially denoised using Gaussian Blur^36^ during the process. Denoising the pictures aids in lowering the histopathological image noise. The SigmaX and SigmaY parameters are set to 90 and 90, respectively, during denoising. The images are initially resized from their original dimensions of (1728, 2304) to (64, 64, 3). This resizing serves multiple purposes. Firstly, it enhances the processing efficiency of our computational environment, allowing for quicker and smoother image processing. Furthermore, downsizing the images facilitates the training of a larger dataset, which is beneficial for improving the robustness and performance of our neural network models. Following this resizing, the images are normalized and subsequently fed into the neural network architectures for further analysis.

**Figure 2:**
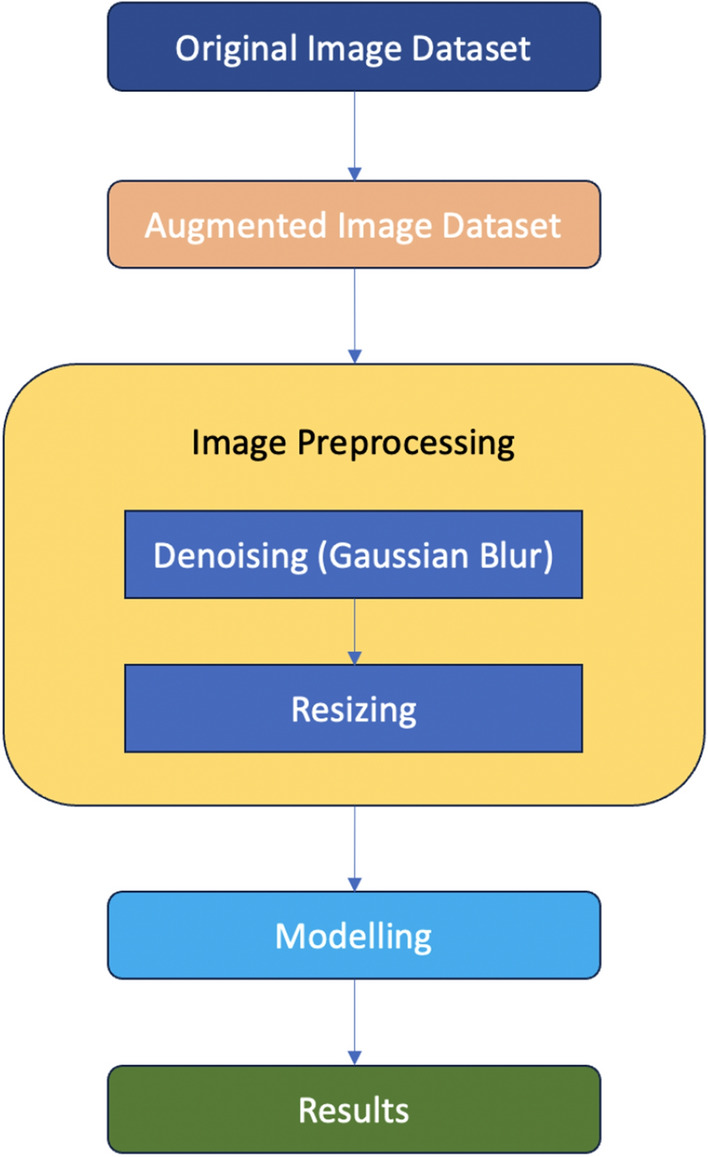
Image Preprocessing. The sequence and steps carried out while preprocessing the images in this work is summarised in graphical format.

Figure 2 summarizes the image preprocessing techniques undertaken in this study.

### Approaches

The current work is mostly broken down into two distinct segments. A unique CNN architecture is suggested in the initial phase for the diagnosis of cholangiocarcinoma. This architecture's lightweight design does not sacrifice diagnostic performance. This would result in the creation of a more trustworthy AI-based system for the diagnosis of cholangiocarcinoma because the model's diagnostic performance is not impacted. During the subsequent phase of this work, a number of well-known existing CNN architectures using transfer learning were also taken into consideration to get a better understanding of how well the proposed architecture performed. A visual illustration of the methods we used during this research project is shown in Figure 4.

#### Proposed CNN Architecture–CholangioNet

*CholangioNet*–a novel lightweight deep learning architecture^39^ is proposed to automate the diagnosis of Cholangiocarcinoma. The proposed architecture is designed to be lightweight without compromising the performance of the AI model. The proposed architecture is developed in such a way that the performance of the architecture can easily outperform the performance of existing state of art works in this domain.

*CholangioNet*–the suggested lightweight architecture, is composed of convolution layers, max-pooling layers for image feature extraction. The fully connected layers placed towards the end of the architecture were on the other hand used for image classification. The architecture uses softmax activation function to facilitate the diagnosis of Cholangiocarcinoma.

The suggested lightweight architecture is intended to accept images with dimensions of (64, 64, 3). A convolution layer with 8 filters is presented as the initial layer of this design. This is followed by two more convolution layers having 32 and 128 filters respectively. The max-pooling layer with a stride 2 receives the layer's output. The output of this max-pooling layer is again fed to a convolution layer having 256 filters. This is followed by a max-pooling layer. The stride of this maximum pooling layer is 2.

**Figure 3:**
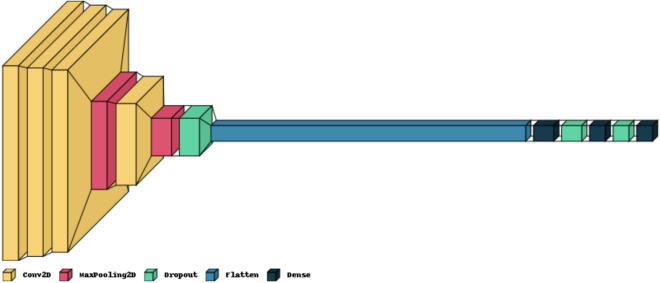
Proposed *CholangioNet* architecture.

The output is finally fed into a series of dense layers to enable image classification. We have used ReLU^31^ as the activation function. We have finally used softmax classifier^37^ in the final dense layer to enable the classification task. The architecture is also supported by Adam Optimizer to enable enhanced model learning. Dropout layers were also added to prevent the problem of overfitting during the training process. Equation (1) corresponds to the equation of ReLU activation function used in this architecture design. in this Figure 3 provides a pictorial representation of the proposed neural network architecture, *CholangioNet.* Table 3 summarizes the hyperparameters that were used in the proposed architecture.

1 $$ReLU\left( x \right) = {\text{max}}\left( {0,\;x} \right)$$

**Table 3:** Hyperparameters of *CholangioNet*.

Hyperparameters	Values
Number of convolution layers	4
Number of max-pool layers	2
Activation function	ReLU and Softmax
Optimizers	Adam
Learning rate	0.001

#### Transfer Learning

On the basis of this transfer learning comparison, we compare the outcomes of *CholangioNet* to those of several well-known contemporary architectures and make judgements regarding the suggested lightweight deep learning design. Transfer Learning^32^ is a technique that allows AI models that have been learned for one task to be applied to another. In this procedure, we employed ImageNet weights.

VGG16^33^: This CNN architecture was created in 2014 by K. Simonyan and A. Zisserman. Deep neural network with 16 layers is this architecture. 13 convolutional layers and 3 fully connected layers make up this architecture. Smaller 3 × 3 receptive fields throughout its network set it apart from and enhance previously well-known architectures like AlexNet. Its decision functions are more discriminative since it has two non-linear activation functions.

VGG19^34^: This well-known 19-layer deep neural network design was created by Simonyan and Zisserman. It has three fully connected layers and 16 convolution layers. Strides are set to 1 in this architecture, and filters (3,3) are used. The (2, 2) max-pooling layers are also used.

ResNet50^35^: This is yet another deep neural network architecture. Kaiming et al. created this architecture in 2015. This architecture has skip connections and 50 layers. The skip connections assist this architecture in overcoming the significant vanishing gradient issue that plagues many CNNs. In this architecture, the 2 layer block from ResNet34's predecessor ResNet34 is swapped out for a 3 layer block.

ResNet101^36^: This neural network design is incredibly deep and well-known. There are 101 layers in this architecture. It consists of numerous 3 layer blocks, batch normalisation layers, and convolution layers. It is important to note that ResNet101 is less difficult than VGG16 and VGG19 despite having a highly deep neural network design.

**Figure 4:**
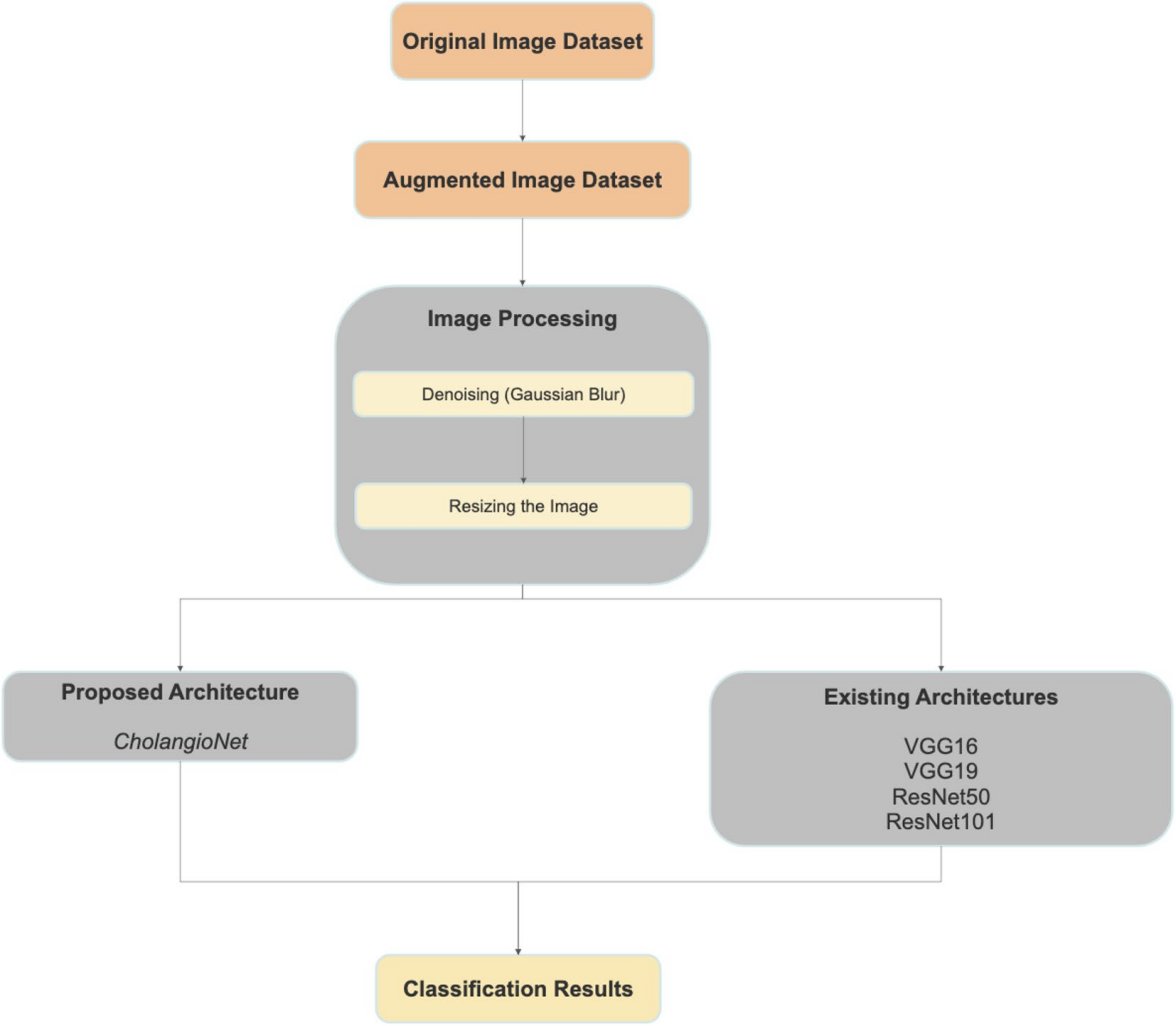
Overall Workflow. This diagram summarizes the sequence of steps that were undertaken while carrying out this entire work. This started with augmenting the original image dataset, followed by the application of minimal preprocessing techniques like denoising and resizing of images. This followed the training of the models that were taken into consideration in this work. Finally, the performance of the architectures that were considered in this study were compared and analyzed.

The overall workflow carried out throughout this effort is summarised in Fig. 4.

## Results and discussion

The experiments executed out as a part of this work was carried out in google colab environment. The capability of the GPU offered by the Google Colab was also used to demonstrate the current work. This made it possible to process the enormous volume of image data more quickly.

The outcomes of the current work were mostly developed using the Python programming language. Several deep learning and data augmentation based libraries were used throughout the project at various stages. In order to boost the quantity of data instances, ImageGenerator was utilised throughout the Data Augmentation procedure. In the current work, libraries like Tensorflow and Keras were also utilised. It is important to note that various libraries, including OpenCV, Numpy, and Matplotlib, were utilised at various stages in this study work.

In the first part of the project, *CholangioNet*, a novel deep learning architecture, is created for the diagnosis of cholangiocarcinoma. The initial architecture of the neural network used to diagnose cholangiocarcinoma was one that was relatively deeper. However, it was discovered that this architecture performed poorly. As a result, the network was adjusted to enhance its ability to diagnose cancer with the highest degree of dependability. The network was altered, but the goal of making it lightweight was retained. In essence, this was accomplished by using a relatively simple deep neural network and fewer model parameters. As a result, the suggested *CholangioNet* architecture for the diagnosis of Cholangiocarcinoma was eventually developed.

The full Cholangiocarcinoma image dataset, which included 15,000 images, was then divided into training and testing sets using an 80:20 ratio. As a consequence, a testing set contained 3000 photographs, whereas the training set contained 12000 images. Utilising images from the training set, deep learning models were trained. The performance of the deep learning models on new data was verified using the testing set of this dataset.

In this study, it is pertinent to note that the total number of parameters of the *CholangioNet* was determined to be 9M+. The total number of parameters of VGG16, VGG19, ResNet50 and ResNet101 on the other hand, was observed to be 14M+, 20M+, 24M+ and 43M+. As clearly observed the proposed architecture has lesser number of parameters in comparison with the other architectures that were taken into consideration in this study, making the proposed architecture lightweight in nature. It is also worth noting that the number of layers present in VGG16, VGG19, ResNet50 and ResNet101 corresponds to 16, 19, 50 and 101 layers respectively. This indicates that the existing architectures are very deep neural network architectures. However, the proposed architecture has just 4 convolution layers, 2 max pooling layers and 3 dense layers, indicating that the proposed architecture is relatively less deep than the prominent existing architecture that were taken into consideration in this study, making it a relatively less deep neural network architecture.

Accuracy, Loss, Precision, and Sensitivity measures are mostly used in the current work to assess how well deep learning models performed.

How accurately a model can categorise the data points is measured by its accuracy. It is described as the proportion of accurate forecasts to all of the model's predictions. Equation (2) corresponds to the mathematical formula for calculating accuracy.

2 $$Accuracy \% = \frac{{True\;Positive \;\left( {TP} \right) + True\;Negative\; \left( {TN} \right)}}{{True\;Positive\;\left( {TP} \right) + False\;Positive \;\left( {FP} \right) + True\;Negative\;\left( {TN} \right) + False\;Negative \;\left( {FN} \right)}} *100$$

Precision is a metric for determining the proportion of positive class predictions that are correct. Equation (3) corresponds to mathematical equation for calculating precision.

3 $$Precision \;\% = \frac{{True\;Positive\; \left( {TP} \right)}}{{True\;Positive\;\left( {TP} \right) + False\;Positive\; \left( {FP} \right)}} * 100$$

Sensitivity is defined as a measure to determine how accurately a model is able to detect the positive samples. It is also known as recall. Equation (4) corresponds to the mathematical formula for calculating sensitivity/recall.

4 $$Sensitivity/Recall \;\% = \frac{{True\;Positive \left( {TP} \right)}}{{True\;Positive\;\left( {TP} \right) + False\;Negative\; \left( {FN} \right)}} * 100$$

It is pertinent to note that Loss metrics was also used to gain better insights into the learning curve of deep learning architecture.

Table 4 summarizes the performance of the proposed lightweight deep learning architecture, *CholangioNet*, presented in the present work.

**Table 4:** Comparison of Performance Metrics for lightweight *CholangioNet.*

Performance metrics	Epochs			
	50	100	150	200
Accuracy (max. %) Validation accuracy (max. %)	99.35 98.10	99.50 98.40	99.84 98.56	99.90 98.40
Precision (max. %) Validation precision (max. %)	100 100	99.97 100	100 100	100 100
Recall (max. %) Validation recall (max. %)	99.97 100	100 100	100 100	100 100
Loss (min.) Validation loss (min.)	0.0249 0.0901	0.0249 0.0882	0.0249 0.0794	0.0037 0.0941

*CholangioNet*, the proposed lightweight neural network architecture, is trained on the whole image dataset, which has 15000 images in total. The model is trained on 80 percent of the data and then put to the test on the remaining 20 percent. Accuracy, Precision, Sensitivity, and Loss are used to evaluate the effectiveness of the suggested model.

As observed from Table 4, the proposed lightweight *CholangioNet* architecture was initially trained for 50 epochs. Maximum training accuracy for the architecture was determined to be 99.35%, and maximum testing accuracy was found to be 98.10%. The maximum precision on the other hand recorded 100% on both the training and testing sets of the data. The maximum testing recall recorded a value of 100% and the maximum training recall on the other hand recorded 99.97%. It is worth noting that the minimum losses recorded for the training and testing sets of the data was found to be 0.0249 and 0.0901 respectively. Detailed analysis on the learning curves of the *CholangioNet* at 50 epochs revealed a display of satisfactory learning curve. Hence, we next trained the proposed *CholangioNet* for 100 epochs.

The maximum training accuracy recorded 99.50% and testing accuracy recorded 98.40% when lightweight *CholangioNet* was trained for 100 epochs. On both the training and test data sets, accuracy reached a maximum of 100%. Similarly, the maximum training and testing recall recorded 100%. It is also worth noting that the minimum losses recorded on the data's training and testing sets are 0.0170 and 0.0882, respectively.

As observed, on increasing the number of training epochs, the proposed *CholangioNet* displayed a better performance. Hence, we next trained the proposed network for 150 epochs.

After training the proposed lightweight neural network *CholangioNet* for 150 epochs, the maximum training and testing accuracy were 99.84% and 98.56%, respectively. On the other hand, the maximum training recall recorded 100% and the maximum testing 100%. It was also determined that the architecture achieved a perfect 100% accuracy on both the testing and training data sets. On the training and testing sets, the minimum losses were 0.0057 and 0.0794, respectively. Looking at the improved performance of the proposed architecture for the diagnosis of the carcinoma, we next trained the proposed architecture for 200 epochs.

We trained the proposed lightweight *CholangioNet* for 200 epochs. Maximum training and testing accuracy were 99.90% and 98.40%, respectively. The precision and recall on the other hand recorded training accuracy of 100% and testing accuracy of 100%. However, for the training and testing data sets, the smallest losses were 0.0037 and 0.0941.

**Figure 5:**
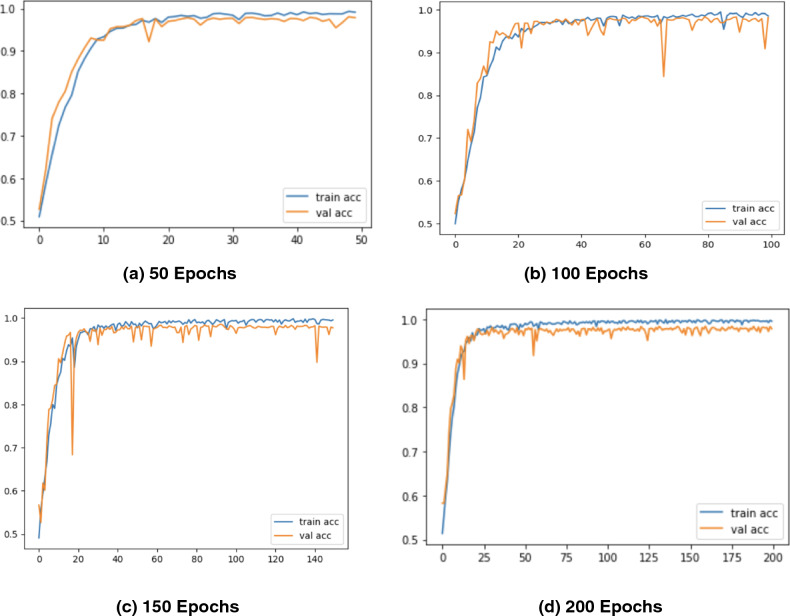
Accuracy Graphs of the Proposed *CholangioNet* architecture. (**a**) proposed model run for 50 iterations (**b**) proposed model run for 100 iterations (**c**) proposed model run for 150 iterations (**d**) proposed model run for 200 iterations (**e**) comparison of accuracy graphs on various epochs.

**Figure 6:**
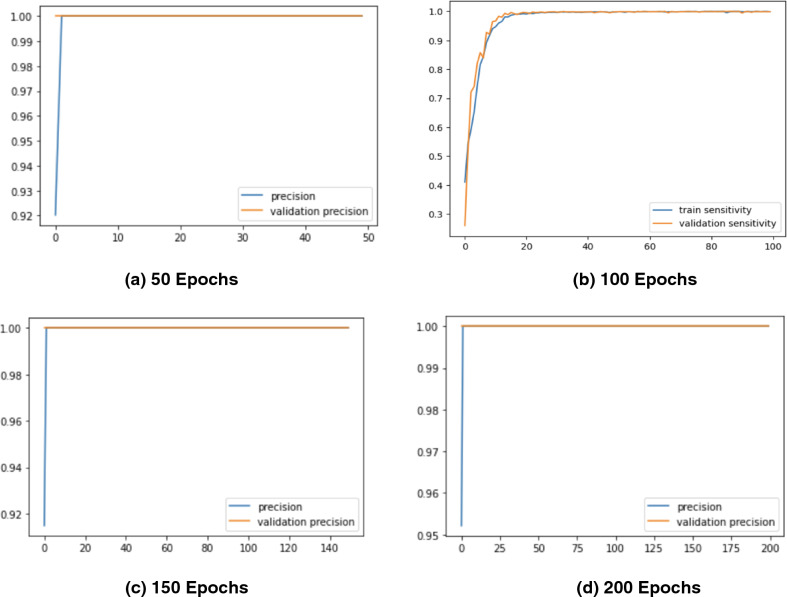
Precision Graphs of the Proposed *CholangioNet* architecture. (**a**) proposed model run for 50 iterations (**b**) proposed model run for 100 iterations (**c**) proposed model run for 150 iterations (**d**) proposed model run for 200 iterations (**e**) comparison of precision graphs on various epochs.

**Figure 7:**
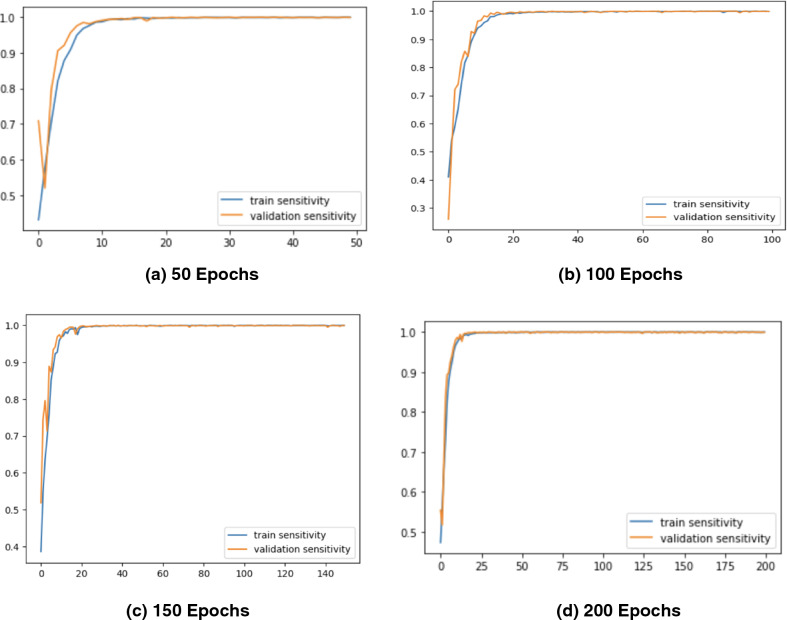
Sensitivity Graphs of the Proposed *CholangioNet* architecture. (**a**) proposed model run for 50 iterations (**b**) proposed model run for 100 iterations (**c**) proposed model run for 150 iterations (**d**) proposed model run for 200 iterations (**e**) comparison of sensitivity graphs on various epochs.

As observed that with an increase in the number of epochs, the proposed *CholangioNet* is found to display good diagnostic performance results. The generation of such extremely good results on the validation set, indicated the better efficiency of the proposed lightweight *CholangioNet* for the diagnosis of Cholangiocarcinoma. The development of such a highly reliable and efficient model, resulted in the evolution of a high performing AI based system. The accuracy, precision, sensitivity, and loss graphs for 50, 100, 150 and 200 epochs are shown in Figure 5, 6, 7, 8. Further, performance comparison graphs for accuracy, precision, sensitivity and loss metrics are shown in Figure 9.

**Figure 8:**
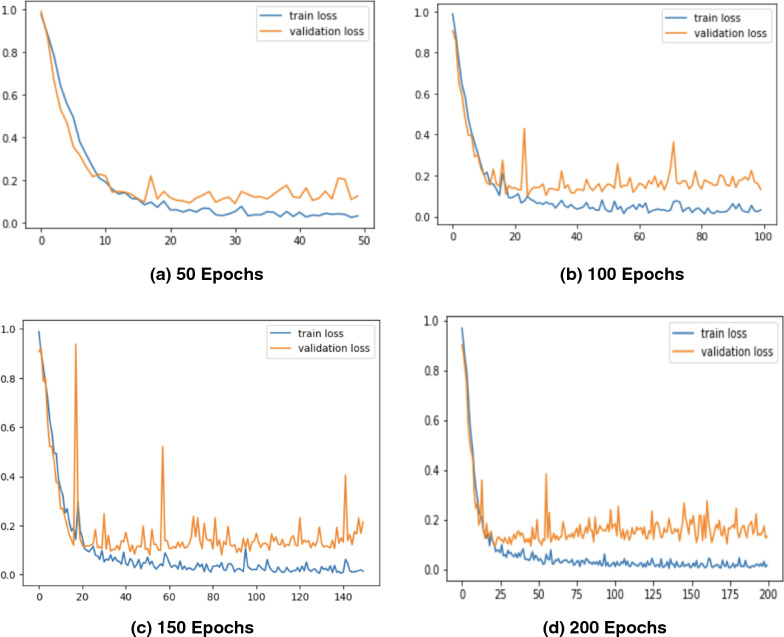
Loss Graphs of the Proposed *CholangioNet* architecture. (**a**) proposed model run for 50 iterations **(b)** proposed model run for 100 iterations (**c**) proposed model run for 150 iterations (**d**) proposed model run for 200 iterations.

**Figure 9:**
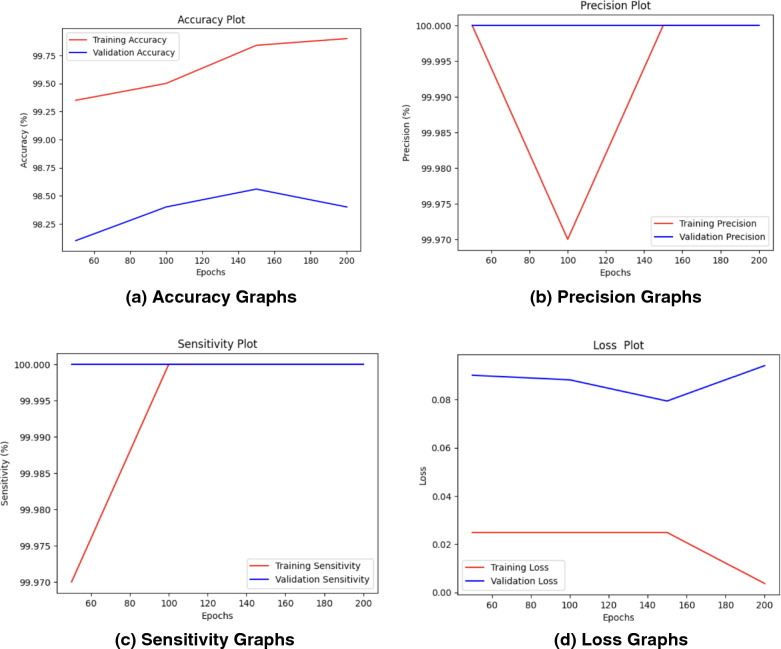
Performance Comparison Graphs of the Proposed *CholangioNet* architecture. (**a**) comparison of accuracy graphs on various epochs (**b**) comparison of precision graphs on various epochs (**c**) comparison of sensitivity graphs on various epochs (**d**) comparison of loss graphs on various epochs.

In the next phase of the present work, we studied and compared the performance of the prominent existing architectures like VGG16, VGG19, ResNet50 and ResNet101 using transfer learning with the proposed architecture *CholangioNet*. This would enable us to gain better insights into the performance of the proposed *CholangioNet.* To begin with we train the VGG16 network using transfer learning for 50 epochs and analyze its performance. Table 5 summaries the performance metrics of the prominent existing VGG16 architecture for the diagnosis of Cholangiocarcinoma.

**Table 5:** Performance Metrics of VGG16 using transfer learning.

Performance metrics	Epochs			
	50	100	150	200
Accuracy (max. %) Validation accuracy (max. %)	95.37 96.23	97.18 97.10	97.73 97.46	98.20 97.69
Precision (max. %) Validation precision (max. %)	100 100	100 100	100 100	100 100
Recall (max. %) Validation recall (max. %)	99.55 99.83	99.79 100	99.87 100	99.93 100
Loss (min.) Validation loss (min.)	0.1267 0.1451	0.0842 0.1313	0.0647 0.1153	0.0537 0.1207

Table 5 demonstrates that the VGG16 architecture attained a maximum training accuracy of 95.37% and a maximum testing accuracy of 96.23% after 50 training iterations. On the other hand, the highest accuracy was 100% on both the data sets used for training and testing. Maximum recalls for training and testing were recorded at 99.55% and 99.83%, respectively. It is important to note that the data's training and testing sets' minimal losses were found to be 0.1267 and 0.1451, respectively. VGG16 was trained for 100 epochs because it was discovered to produce good outcomes.

The network's maximum training and testing accuracy were 97.18% and 97.10%, respectively, after 100 interations of transfer learning training. Maximum recalls throughout training and testing were 99.79% and 100%, respectively. On the other hand, the precision recorded 100% on both the training and testing data sets. We saw an improvement in the VGG network's performance with the smallest losses recording 0.0842 and 0.1313 on the training and testing sets of the data. So, we then trained this network for 150 iterations using transfer learning.

When VGG16 was trained for 150 epochs utilizing transfer learning ideas, the maximum training and testing accuracy were 97.73% and 97.46%, respectively. The maximum training recall and the maximum testing recall recorded 99.87% and 100% respectively. The architecture was also found to display a maximum training precision and a maximum testing precision of 100% and 100% respectively on the training and testing sets of the data. The minimum losses on the training and testing sets recorded 0.0647 and 0.1153 respectively. We next trained this architecture for 200 epochs.

The maximum training accuracy for VGG16 was 98.20%, while the maximum testing accuracy was 97.69% after 200 cycles of training using transfer learning principles. On the other hand, the precision recorded 100% on both the training and testing data sets. The highest recall rates for training and testing were 99.93% and 100%, respectively. On the other hand, on the training and testing sets of the data, the minimum losses were 0.0537 and 0.1207, respectively.

As seen, we discover that the VGG16 architecture performed better after 200 epochs of training. After examining the performance of the VGG16, we proceed to examine the performance of the VGG19 utilizing transfer learning ideas. The performance characteristics of the well-known current VGG19 architecture for the diagnosis of cholangiocarcinoma are summarized in Table 6.

**Table 6:** Performance Metrics of VGG19 using transfer learning.

Performance metrics	Epochs			
	50	100	150	200
Accuracy (max. %) Validation accuracy (max. %)	93.25 95.06	94.51 95.46	95.55 96.66	96.40 97.03
Precision (max. %) Validation precision (max. %)	100 100	100 100	100 100	100 100
Recall (max. %) Validation recall (max. %)	98.37 99.46	98.83 99.56	99.26 99.86	99.58 99.86
Loss (min.) Validation loss (min.)	0.1854 0.1612	0.1577 0.1430	0.1238 0.1240	0.1018 0.1200

When first trained using transfer learning for 50 epochs, the VGG19 architecture achieved the maximum training accuracy of 93.25% and the maximum testing accuracy of 95.06%. On the other hand, the maximum precision recorded 100% on both the testing and training sets. The maximum training recall and the maximum testing recall recorded a value of 98.37% and 99.46% respectively. It is worth noting that the minimum losses recorded for the testing and training sets of the data was found to be 0.1612 and 0.1854 respectively. Since, VGG19 was found to generate good performance, the network was next trained for 100 epochs.

After training the neural network for 100 epochs with transfer learning, we observed the maximum training accuracy recorded 94.51% and the maximum testing accuracy recorded 95.46%. The maximum training recall and maximum testing recall recorded a 98.83% and 99.56% respectively. The precision on the other hand recorded 100% on both testing and training sets of the data. With the minimal losses recording 0. 1430 and 0. 1577 on the testing and training sets, we observed an improved performance of the VGG network. Hence, this network was then trained for 150 epochs.

When VGG19 was trained for 150 epochs utilizing transfer learning ideas, the maximum training and testing accuracy were 95.55% and 96.66%, respectively. The maximum training recall and the maximum testing recall recorded 99.26% and 99.86% respectively. On the testing and training sets of data, the architecture was determined to have the maximum testing precision and the maximum training precision of 100% and 100%, respectively. The minimum losses on the testing and training sets recorded 0.1240 and 0.1238 respectively. We next train this architecture for 200 epochs.

On training the network for 200 epochs, the maximum training accuracy and the maximum testing accuracy recorded 96.40% and 97.03% respectively. The precision on the other hand recorded 100% on both training and testing sets of the data. The maximum training recall and the maximum testing recall recorded 99.58% and 99.58% respectively. The minimum losses on the other hand recorded 0.1018 and 0.1200 on the training and testing sets of the data respectively.

As observed, we find that the VGG19 architecture has performed better when found to be trained for 200 epochs. Having analyzed the performance of the VGG19, we next analyze a much deeper neural network architecture, ResNet50 using the concepts of transfer learning. Table 7 summaries the performance metrics of the prominent existing ResNet50 architecture for the diagnosis of Cholangiocarcinoma.

**Table 7:** Performance metrics of ResNet50 using transfer learning.

Performance metrics	Epochs			
	50	100	150	200
Accuracy (max. %) Validation accuracy (max. %)	47.34 49.43	50.00 55.19	51.77 57.06	52.65 55.13
Precision (max. %) Validation precision (max. %)	100 100	100 100	100 100	100 100
Recall (max. %) Validation recall (max. %)	20.91 27.55	35.51 40.12	48.81 61.25	47.47 57.61
Loss (min.) Validation loss (min.)	0.9766 0.9978	0.9589 0.9359	0.0647 0.1153	0.9478 0.9318

When first trained with transfer learning for 50 epochs, the ResNet50 architecture achieved the maximum training accuracy of 47.34% and the maximum testing accuracy of 49.43%. On the other hand, the maximum precision recorded 100% on both the testing and training sets of the data. The maximum testing recall and the maximum training recall recorded a value of 27.55% and 20.91% respectively. It is worth noting that the minimum losses recorded for the testing and training sets of the data was found to be 0.9978 and 0.9766 respectively. As observed, the performance of ResNet50 using the concepts of transfer learning is found to generate poor performance results when trained for 50 epochs. Hence, with a hope that the performance of this architecture would increase with an increase in the number of epochs, we train ResNet50 using transfer learning for 100, 150 and 200 epochs. To begin with, we analyze the performance of the network when trained for 100 epochs.

After training the neural network for 100 epochs using the concepts of transfer learning, we found that the maximum training accuracy recorded 50% and the maximum testing accuracy recorded 55.19%, respectively. The maximum training recall and maximum testing recall recorded a 35.51% and 40.12% respectively. The precision on the other hand recorded 100%. With the minimal losses recording 0.9359 and 0.9589 on the testing and training sets of the data, we observed a slight improvement in the efficiency of the network when compared with the results obtained when the network was trained for 50 epochs. As a result, we train the network for 150 epochs next.

When Resnet50 was trained for 150 epochs utilizing transfer learning concepts, the maximum training and testing accuracy were 51.77% and 57.06%, respectively. Maximum training precision and maximum testing precision of 100% were also observed for the architecture. The maximum training recall was 48.81%, and the greatest testing recall was 61.25%. The minimum losses on the training and testing sets recorded 0.0647 and 0.1153 respectively. Having observed a slight improvement in the performance of the architecture, we next train this architecture for 200 epochs.

On training the network for 200 epochs, the maximum training accuracy recorded 52.65% and the maximum testing accuracy recorded 55.13%. The precision on the other hand recorded 100% on both testing and training sets of the data. The maximum training recall and the maximum testing recall recorded 47.47% and 57.61% respectively. The minimum losses on the other hand recorded 0.9318 and 0. 9478 on the testing and training sets of the data respectively.

As observed, ResNet50 is not found to display good performance for the development of a reliable AI based system for the diagnosis of Cholangiocarcinoma. We next try to experiment the performance of ResNet101, a much deeper neural network architecture, using the concepts of transfer learning.

Table 8 summaries the performance metrics of the prominent existing ResNet101 architecture for the diagnosis of Cholangiocarcinoma.

**Table 8:** Performance metrics of ResNet101 using transfer learning.

Performance metrics	Epochs			
	50	100	150	200
Accuracy (max. %) Validation accuracy (max. %)	40.40 45.10	41.55 46.66	41.68 47.83	44.94 48.26
Precision (max. %) Validation precision (max. %)	98.40 100	100 100	100 100	100 100
Recall (max. %) Validation recall (max. %)	6.93 8.65	8.75 10.21	9.05 10.11	15.25 17.74
Loss (min.) Validation loss (min.)	1.0642 1.0412	1.0577 1.0330	1.0544 1.0234	1.0302 1.0102

When initially trained with transfer learning for 50 epochs, the ResNet101 architecture achieved the maximum training accuracy of 40.40% and testing accuracy of 45.10%. On the other hand, the maximum precision was 98.40% and 100% on the training and testing data sets, respectively. The maximum training recall and the maximum testing recall recorded a value of 6.93% and 8.65% respectively. It is worth noting that the minimum losses recorded for the testing and training sets of the data was found to be 1.0412 and 1.0642 respectively. As observed, the performance of ResNet101 using the concepts of transfer learning is found to generate very poor performance results when trained for 50 epochs. Hence, with a hope that the performance of this architecture would increase with an increase in the number of epochs, we train ResNet101 using transfer learning for 100, 150 and 200 epochs. To begin with, we analyze the performance of the neural network when trained for 100 epochs.

After training the neural network for 100 iterations using transfer learning, we observed the maximum training accuracy of 41.55% and the maximum testing accuracy of 46.66%. The maximum training recall and maximum testing recall recorded 8.75% and 10.21% respectively. The precision on the other hand recorded 100%. With the minimal losses recording 1.0330 and 1.0557 on the testing and training sets of the data, we observed that there is negligible improvement in the performance of the of the network when compared with the results achieved when the network was trained for 50 iterations.

When Resnet50 was trained for 150 epochs utilizing transfer learning concepts, the maximum training and testing accuracy were 41.68% and 47.83%, respectively. The architecture was also shown to have the maximum training precision and the maximum testing precision of 100% and 100%, respectively. The maximum training recall and the maximum testing recall recorded 9.05% and 10.11% respectively. The minimum losses on the testing and training sets recorded 1.0234 and 1.0544 respectively.

After training the network for 200 epochs, the highest training and testing accuracy were 44.94% and 48.26%, respectively. The precision on the other hand recorded 100% on both training and testing sets of the data. The maximum training recall and the maximum testing recall recorded 15.25% and 17.74% respectively. The minimum losses on the training and testing sets recorded 1.0302 and 1.0102 respectively.

As observed, ResNet101 is not found to display good performance for the development of a reliable AI based system for the diagnosis of Cholangiocarcinoma. Since, all the architectures were found to display better performance at 200 epochs, we compare the performance of the existing architectures, namely, VGG16, VGG19, ResNet50 and ResNet101, with *CholangioNet* at 200 epochs.

Table 9 compares the performance of the existing architectures namely, VGG16, VGG19, ResNet50 and ResNet101 with the proposed architecture *CholangioNet.*

**Table 9:** Comparison of performance metrics (200 Epcohs).

Performance metrics	Epochs				
	VGG16	VGG19	ResNet50	ResNet101	*CholangioNet*
Accuracy (max. %) Validation accuracy (max. %)	98.20 97.69	96.40 97.03	52.65 55.13	44.94 48.26	99.90 98.40
Precision (max. %) Validation precision (max. %)	100 100	100 100	100 100	100 100	100 100
Recall (max. %) Validation recall (max. %)	99.93 100	99.58 99.86	47.47 57.61	15.25 17.74	100 100
Loss (min.) Validation loss (min.)	0.0537 0.1207	0.1018 0.1200	0.9478 0.9318	1.0302 1.0102	0.0037 0.0941

As observed from Table 9, among all the prominent existing architectures trained using the concepts of transfer learning, VGG16 is found to display the best performance for the diagnosis of Cholangiocarcinoma. It is noted that the performance of the VGG architectures is found to be better than the performance of the ResNet architectures taken into consideration. It is also pertinent to note in this context that as the architectures gets deeper and deeper, the performance of the architecture is dropping drastically. However, when the performance of VGG16 is compared with the acheivements of the proposed neural network architecture, it is observed that the AI system developed using *CholangioNet* would prove to be more efficient and reliable than an AI system developed using VGG16. As a result, it is possible to infer that the performance of the suggested neural network architecture, *CholangioNet*, is rather suitable for the construction of a dependable and efficient AI-based system for the diagnosis of Cholangiocarcinoma.

Finally, we evaluate *CholangioNet*, the suggested architecture, against the current state of the art in this field. The current state of the art is summarized in Table 10.

**Table 10:** Comparison of performance of *CholangioNet* with the existing works.

Research paper	Performance comparison
6	Model accuracy *without* spectral interval convolution (with HSI image) ResNet – 85% (78.5%) InceptionNet – 85.3% (75.2%) Model accuracy with spectral interval convolution (with HSI data) ResNet – 88.2% InceptionNet – 87.9%
7	The AI model achieved 88.5% accuracy on the internal validation sets and 84.2% on the independent test sets. When the model was correct, the aid increased accuracy across the board for all levels of pathologist experience, regardless of case difficulty. When the model was wrong, however, the assistance dramatically reduced accuracy across the board for all levels of pathologist experience
8	The model corresponding to the detection of Differentiation Degree recorded an average accuracy of 71% and 69.9% on testing and training sets respectively The model corresponding to the detection of Lymph Node Metastasis was found to record an average accuracy of 81.4% and 83.3% on the training and testing sets respectively
9	The proposed model for binary classification achieved an average accuracy of 82.5% while for multiclass classification it achieved an average accuracy of 73.4%
10	Liver Segmentation accuracy – 97.72% Tumor Segmentation accuracy – 98.7% The proposed ELM method recorded an accuracy of 96.56%, sensitivity of 94.33%, and specificity of 97.78%
11	The proposed model recorded an AUC score of 0.72. The specificity accuracy and sensitivity, and recorded 100%, 69.70% and 44% respectively
12	The proposed algorithm could record a recognition accuracy of 88% on the private dataset used in this study
13	Diagnostic Accuracy for one input model and two input model corresponded to 60% and 61% respectively. On the other hand, Radiologist1 and Radiologist2 recorded an accuracy of 55% and 53% respectively
14	The proposed model recorded an accuracy of 92%. It also recorded a sensitivity and specificity of 92% and 98% respectively
15	This system recorded an 82.9% sensitivity
16	The proposed system worked well. The system recognized the significance of the Hepatobiliary tract during liver SBRT on its own
17	The Radiomics model achieved 78.7% accuracy, 84.6% sensitivity, and 77.1% specificity, respectively. The Logistics Model, on the other hand, achieved 59% accuracy, 69.2% sensitivity, and 56.2% specificity. The sensitivity, specificity, and accuracy Combined Model were 92.3%, 70.8%, and 75.4% respectively
18	The highest accuracy recorded for three class classification corresponds to 90.22% for slice and 94.92% for patient level. While the highest accuracy recorded for two class classification corresponds to 94.17% and 97.44% for slice and patient level respectively
19	The proposed model was found to generate good results
20	The model could classify the normal and tumor images with 94% accuracy on the test set of the image dataset They were also able to detect the tumor images from a large pool of images containing common biliary disease with 88% accuracy
21	The combination of Random Forest and Linear Discriminant Analysis (LDA) produced the best results recording a 96.9% accuracy and an AUC score of 0.997
22	The median accuracy on the testing set is determined to be 84% The median AUC score to distinguish between A–B and C–E is found to be 0.92
23	MRI radiomics features was found to display better AUC score (highest AUC score: 0.77) in comparison with the CT radiomics features
24	The combination nomogram was found to generate better prediction results than SVM alone
25	Random forest was found to display better results when compared with Support Vector Machine and Deep Neural Network
26	SVM model recorded 0.855 and 0.847 AUC score on training and testing cohorts respectively
27	Random Forest recorded the highest accuracy of 88%
28	The proposed novel Deep Margin Cosine Autoencoder (DMCA) model was found to generate good results
29	Logistic Regression Classifier attained the maximum ROC, recording a value of 0.82
30	The combination of Logistic Regression classifier and Z score standardization recorded an average AUC score of 0.912 and 0.884 on training and testing cohort respectively. While the combination of Z score standardization and Stochastic Gradient Descent based linear regression recorded an average R^2^ of 0.975 on the training cohort
31	ResNet50 was used by the authors in this study. The accuracy, specificity and sensitivity on the validation set of the dataset recorded 96.5%, 94% and 97.8% respectively. Further, the ROC was also determined to be 0.994
Proposed architecture (*CholangioNet*)	The maximum training accuracy recorded 99.90% and the maximum testing accuracy recorded 98.40%, respectively Both the precision and the recall recorded 100% on both testing and training sets of the data

The work carried out in^6^ essentially emphasizes on the advantage of diagnosing diseases using HSI data. They concluded that using HSI data can significantly improve the diagnostic performance. However, in this context it is worth noting that generating/procuring HSI data is expensive, complex and time consuming. Hence, it might not always be possible to generate HSI data for diagnostic purposes. Thus, it becomes important to develop an AI based model that provides reliable diagnostic results using RGB images. When we compare the performance of the proposed architecture, *CholangioNet*, with the performance of the architecture used for diagnosis in^6^, it is found that the diagnostic performance of *CholangioNet* using RGB images is way better than the diagnostic performance of architecture used in^6^ on HSI data. The work carried out in^7^ emphasized on the development of reliable AI based diagnostic systems, since the diagnostic performance of the radiologist seems to be affected by the inputs received from the AI based diagnostic systems. Hence, there becomes a dire requirement to develop high performing diagnostic systems. The various diagnostic performances achieved by the works carried out in^8–30^ is also analysed. In^8^, the detection accuracy of differentiation degree was observed to be 71% on the training set, while the detection accuracy of the lymph node metastasis was found to be 83.3%. Similarly, in^9^, the binary classification and multiclass classification recorded an aacuracy of 82.5% and 73.4%. Though the accuracy of classification of quite good in both^8,9^, however, it would have been better if the performance of the AI model could have been improved further, in view of the observations made from^7^. In^10^, the proposed ELM method recorded an accuracy of 96.56%, sensitivity of 94.33%, and specificity of 97.78%. The work carried out in^11^ on the other hand recorded a specificity, accuracy and sensitivity recorded 100%, 69.70% and 44% respectively. Similarly, the work carried out in^12^, recorded 88% recognition accuracy using the proposed model. In^13^, the diagnostic accuracy for one input model and two input models corresponded to 60% and 61% respectively. Wang et al.^15^, on the other hand recorded 82.9% sensitivity. While the work performed in^14^, recorded an accuracy, sensitivity and specificity of 92%, 92% and 98% respectively. The radiomics model experimented in^17^, achieved an accuracy of 78.7%. The Logistics model recorded an accuracy of just 59%. While combined model recorded an accuracy of 74.5%. The work performed in^18^ had performed a three class classification and recorded the highest accuracy of 90.22% and 94.92% respectively. The work performed in^20^ also displayed performance by recording an accuracy of 94%. While the experimentation executed in^21,22^, recorded an AUC score greater than 0.90. The work carried out in^23–26^, also recorded good results. In^27^, Random forest recorded the highest accuracy of 88%. While the work carried out in^29,30^, also displayed good results. The experimentation carried out in^31^, on the other hand also recorded an accuracy, sensitivity and specificity of 96.5%, 94% and 97.8% respectively on the validation part of the dataset. Though the existing state of the art displayed good performance in diagnosing the disease, however, a further improvement in the performance of the AI model would enable the existance of a more reliable AI based model, which is required in view of the observations made from^7^. Thus, the proposed architecture, *CholangioNet*, can generate better and more reliable and efficient diagnostic results of Cholangiocarcinoma in comparison with the existing state of the arts.

## Conclusion and future work

The authors of^6^, noted that the performance of the deep learning models gets restricted due to the limited information contained in a RGB images in comparison with the Hyperspectral image. However, it is pertinent to note that the procurement of such hyperspectral images is costly. Hence, there becomes a need to develop an equally effective deep learning model that can produce reliable diagnostic results using RGB images. The present work essentially aims at developing an efficient and reliable AI based system using RGB histopathological images for smart diagnosis of Cholangiocarcinoma. For this, we have used a RGB based histopathological image dataset procured from Kaggle. Upon initial analysis of the image dataset, it was found that the number of data instances in the image dataset was very limited. It is pertinent to note that during real world application of this AI based system, it is quite possible that such histopathological images may not be captured under uniform environmental conditions. Hence, we performed data augmentation to generate images under varying conditions to enable the development of a more universal, reliable and efficient AI based diagnostic system. The features from these images were then enhanced using certain image pre-processing procedures. Thereafter, the enhanced images were fed into the proposed deep learning architecture, *CholangioNet. CholangioNet* is designed to be a lightweight neural network for the diagnosis of Cholangiocarcinoma. The network was designed to be lightweight without compromising the main objective of designing a reliable and efficient AI based diagnostic system. The neural network was made lightweight by designing the network shallower and also having lesser number of parameters to enable faster training of the deep learning architecture. A comparative analysis with the performance of the prominent existing architectures (VGG16, VGG19, ResNet50 and ResNet101) using transfer learning is done to gain further insights into the performance of the proposed architecture *CholangioNet*. The proposed architecture despite being lightweight was found to have more potential than the prominent existing architectures for the development of a reliable AI based diagnostics system. Finally, the performance of the proposed network, *CholangioNet*, was then compared with the existing state of the art for better analysis. The performance of the proposed network was also found to generate better results than the existing state of the arts. Finally, it is concluded that the performance of the proposed architecture is better than the prominent architectures and the existing state of the arts in this domain as discussed above.

It is also concluded that the proposed lightweight neural network, *CholangioNet*, evolved as a highly reliable and efficient AI based diagnostic system for the diagnosis of Cholangiocarcinoma using RGB based histopathological images. However, it is important to note that the current architecture doesn’t segment the tumor regions for better diagnostic evaluations. Hence, this becomes a limitation of this architecture that will be eliminated in future by extending this model to segment the extact tumorous region for a more informed diagnosis.

The present work could also be extended by deploying this proposed lightweight neural network into a web based application to enable faster diagnosis of Cholangiocarcinoma to a wide range of audience in the healthcare industry. The performance of this novel model in diagnosing other types of cancers in the abdominal regions will also be explored in the future.

## Data Availability

You may find the data used to support this study at Kaggle. Website Link: Microscopic Hyperspectral Choedoch Dataset https://www.kaggle.com/datasets/ethelzq/multidimensional-choledoch-database.
